# Commentary on ‘Facing the phase problem’ by Wayne Hendrickson

**DOI:** 10.1107/S2052252523007340

**Published:** 2023-08-24

**Authors:** Janet L. Smith

**Affiliations:** a Life Sciences Institute and Department of Biological Chemistry, University of Michigan, Ann Arbor, MI 48109, USA

**Keywords:** anomalous diffraction

## Abstract

A commentary on Wayne Hendrickson’s article ‘Facing the phase problem’.

The 2023 Ewald Prize honors Wayne Hendrickson for his many contributions to the field of macromolecular crystallography. His monograph, ‘Facing the phase problem’, is a beautifully written short history of crystallographic phasing, with a focus on experimental phasing. It begins in the early years of crystallography, with the dawning recognition that phase determination is a ‘problem’, and includes the contributions of Paul Ewald, for whom the Prize is named.

Wayne’s interest in the phase problem began when he was a PhD student at Johns Hopkins University in the laboratory of Werner Love. He and fellow PhD student Ed Lattman developed a method to represent the probability distribution for the phase of each reflection as four numbers, known as Hendrickson–Lattman or *ABCD* coefficients. Their landmark paper (published in *Acta Cryst. B*, 1970) appeared just after they received PhD degrees. The *ABCD* coefficients provided a simple means to combine phase information from sources as vastly different as isomorphous replacement, partial models, redundancy in the asymmetric unit, molecular replacement, and anomalous scattering. Phase combination became an essential tool of macromolecular crystallography as researchers tackled more challenging molecular systems that yielded crystals of increasingly poor diffraction quality.

Long before the explosive growth of macromolecular crystallography, Wayne continued his work on the phase problem as a postdoc with Jerome Karle at the US Naval Research Laboratory (NRL). There he sought to apply Karle’s direct-methods phasing to protein crystallography. However, he quickly became intrigued with an idea to solve the phase problem solely from the anomalous scattering of a few atoms in the protein crystal. This led to the famous crambin experiment. In a tour de force of data collection, Wayne recorded diffraction data from a crambin crystal using the Cu *K*α emission of a sealed-tube X-ray generator equipped with a four-circle diffractometer and a single-point scintillation detector. The data were punched on paper tape, one reflection at a time, as the tape unspooled onto the floor. Some time during the multi-day experiment, a staff member tasked with cleaning the floor ripped the precious unspooled tape from the punch device but, happily, did not remove it from the lab. Wayne processed the data and developed a method to solve the structure using the anomalous scattering of the six sulfur atoms in crambin. To do this, he started with Jerome Karle’s parameterization of the structure factor equation and formulated an observational equation relating the phase and phase probability for each reflection to the observed anomalous difference. This approach allowed him to combine phase probabilities from anomalous scattering and from the sulfur partial structure (via *ABCD* coefficients!), yielding a lovely interpretable 1.5 Å electron density map for crambin.

Simultaneous with the crambin success, Wayne and NRL colleague John Konnert developed the first widely used software for crystallographic refinement of protein atomic models. Their major innovation was to include elements of protein stereochemistry (well established bond lengths, angles, *etc*.) as observations on an equal footing with diffraction data and to refine the atomic models by least-squares minimization. The Konnert and Hendrickson concept of stereochemical details as observables has been in continuous use since then, although today’s software for refinement is far faster and more robust.

To crystallographers working today, Wayne is best known for his role in the breathtaking transformation of protein crystallography into structural biology, which occurred since the turn of the century. Anomalous scattering was prominent in this transformation. Wayne spurred the development of reliably tunable synchrotron beamlines to enhance the weak phasing signal in most anomalous diffraction experiments. He did this by solving novel structures of high biological impact using anomalous diffraction data from tunable beamlines at several synchrotrons. Ever fearless in deploying new technologies to the aid of protein crystallography, he led the development of a cutting-edge tunable beamline for macromolecular crystallography at the Brook­haven synchrotron. Ever motivated to improve crystallographic methods, Wayne developed the multi-wavelength anomalous diffraction method – with the memorable acronym MAD. For MAD, he extended the phasing formulation of the crambin experiment to multi-wavelength data. Arguably of broadest impact, Wayne envisioned a general method to incorporate seleno­methio­nine (SeMet) in recombinant proteins. The ease of introducing an anomalous scatterer that does not interfere with the native structure or the function of a protein was truly transformative. By 2010, SeMet anomalous scattering had become the favored method for experimental phasing in protein crystallography, and so it remains today. When radiation damage from the bright X-ray beams of undulator sources challenged multi-wavelength experiments, MAD was quickly replaced by single-wavelength anomalous diffraction – with the equally memorable acronym SAD.

Wayne also sought to bring the results of protein crystallography to a broad audience at an opportune time. The Protein Data Bank grew by less than 100 entries per year before 1990 and exploded to nearly 700 entries per year in 1993. Correctly anticipating the rapid growth that followed, Wayne was founding editor of *Current Opinion in Structural Biology* (1991, with Aaron Klug), the annual volume *Macromolecular Structures* (1991–2000, with Kurt Wüthrich), and *Structure* (1993, with Carl Brändén).

Wayne’s seminal contributions to macromolecular crystallography and his enthusiasm for protein structure have attracted many talented trainees to his lab. More than 50 current and former lab members (see Fig. 1 ▸) and many more friends, collaborators and colleagues gathered at the 2022 Hendrickson Reunion Symposium at Columbia University. The symposium followed a joyous reunion party and featured a full day of talks from several trainees among the many who went on to lead successful research groups in structural biology. The event was a fitting recognition of Wayne Hendrickson as a leading light of macromolecular crystallography, both for his development of seminal methods that now make X-ray crystallography a standard structural biology approach and his mentorship in training the next generation of creative scientists who continue to expand the reach of X-ray crystallography into the biological sciences. His article to accompany the Ewald Prize, published in this issue of **IUCrJ** (Hendrickson, 2023 ▸), is a terrific addition to our field.

## Figures and Tables

**Figure 1:**
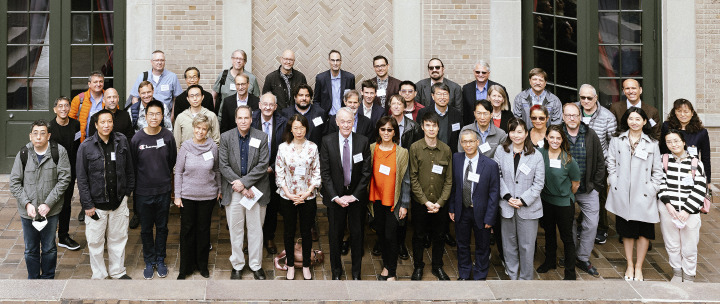
Wayne Hendrickson (front row, center) with former and current members of his research group at the Hendrickson Reunion Symposium, Columbia University, April 2022.
